# Agmatine Protects against Zymosan-Induced Acute Lung Injury in Mice by Inhibiting NF-*κ*B-Mediated Inflammatory Response

**DOI:** 10.1155/2014/583736

**Published:** 2014-08-27

**Authors:** Xuanfei Li, Zheng Liu, He Jin, Xia Fan, Xue Yang, Wanqi Tang, Jun Yan, Huaping Liang

**Affiliations:** State Key Laboratory of Trauma, Burns and Combined Injury, Research Institute of Surgery, Daping Hospital, The Third Military Medical University, Chongqing 400042, China

## Abstract

Acute lung injury (ALI) is characterized by overwhelming lung inflammation and anti-inflammation treatment is proposed to be a therapeutic strategy for ALI. Agmatine, a cationic polyamine formed by decarboxylation of L-arginine, is an endogenous neuromodulator that plays protective roles in diverse central nervous system (CNS) disorders. Consistent with its neuromodulatory and neuroprotective properties, agmatine has been reported to have beneficial effects on depression, anxiety, hypoxic ischemia, Parkinson's disease, and gastric disorder. In this study, we tested the effect of agmatine on the lung inflammation induced by Zymosan (ZYM) challenge in mice. We found that agmatine treatment relieved ZYM-induced acute lung injury, as evidenced by the reduced histological scores, wet/dry weight ratio, and myeloperoxidase activity in the lung tissue. This was accompanied by reduced levels of TNF-α, IL-1*β*, and IL-6 in lung and bronchoalveolar lavage fluid and decreased iNOS expression in lung. Furthermore, agmatine inhibited the phosphorylation and degradation of I*κ*B and subsequently blocked the activation of nuclear factor (NF)-*κ*B induced by Zymosan. Taken together, our results showed that agmatine treatment inhibited NF-*κ*B signaling in lungs and protected mice against ALI induced by Zymosan, suggesting agmatine may be a potential safe and effective approach for the treatment of ALI.

## 1. Introduction

Acute lung injury (ALI) and acute respiratory distress syndrome (ARDS), which is the severest form of injury, are the leading causes of morbidity and mortality in critically ill patients [1]. ALI is characterized by the development of hypoxemia, damage to the alveolar capillary membrane barrier, pulmonary edema, and the resultant respiratory failure [2]. Current therapeutic strategy includes protective ventilation and supportive fluid conservative [3]. Although extensive studies about the pathogenesis of ALI have been conducted, the mortality of ALI remains very high [4]. Thus, it is critical to explore the innovative therapies and effective medications for ALI.

Among the animal models established for the investigation of mechanism involved in ALI, intraperitoneal injection of Zymosan (ZYM) is one of the most commonly used models of ALI [5]. Zymosan is a substance derived from the cell wall of the yeast* Saccharomyces cerevisiae*. When injected into animals, ZYM induces inflammation by a series of mechanisms. Reports show that the onset of ZYM-induced inflammatory response in mouse lung is associated with the gas exchange barrier and that it culminates with maximal neutrophil accumulation, exudate formation, and proinflammatory cytokines production [6, 7]. ZYM is recognized by toll-like receptor 2 (TLR-2) on immune cells (e.g., neutrophils), which subsequently trigger signal cascade for nuclear factor-*κ*B (NF-*κ*B) activation [8]. NF-*κ*B activation is required for maximal expression of many proinflammatory cytokines and chemokines and iNOS involved in the pathogenesis of acute lung injury [9].

Agmatine, a biogenic amine formed by arginine decarboxylation, is widely but unevenly distributed in mammalian tissues. Agmatine has been reported to have various biological actions. It attenuates morphine withdrawal syndromes, inhibits inducible nitric oxide synthase (NOS), and contributes to polyamine homeostasis [10]. Additionally it is known to exert antidepressant, anxiolytic, antitumor cell proliferative and anticonvulsive effects [11]. However, the precise working mechanisms of agmatine are not yet fully understood. The aim of our study was to investigate the protective effects of agmatine on Zymosan-induced acute lung injury and to assess its relative mechanisms.

## 2. Materials and Methods

### 2.1. Reagents

Zymosan and agmatine were obtained from Sigma-Aldrich. Antibodies against iNOS, NF-*κ*B p65, I*κ*B-α, and *β*-actin were obtained from Santa Cruz Biotechnology (Santa Cruz, CA), and those against phosphor (p)-I*κ*B and NF-*κ*B p65 were from Cell Signaling Tech (Danvers, MA). Mouse TNF-α, IL-1*β*, and IL-6 enzyme-lined immunosorbent assay (ELISA) kits were purchased from Boster Biotechnology (Wuhan, China). All suspensions were freshly made before use.

### 2.2. Animals and Treatments

Male C57Bcl/6 mice (weighing 18–22 g) were used in this study. Animal procedures were approved by the Ethics Committee for Animal Experimentation of Third Military Medical University. An inflammation-associated lung injury model was established by aseptic intraperitoneally (IP) injection of ZYM (30 mg/mL suspended in normal Saline (NS)) into mice, at a dose of 600 mg/kg of body weight, as previously described [12]. The same volume of NS was injected through the same route as the sham control.

### 2.3. Histologic Examination

Lungs were harvested for observing morphologic alterations at 24 hrs after ZYM or NS administration. The subjects were fixed with 10% formalin for 8 hrs at room temperature, embedded in paraffin, and sectioned at 5 *μ*m thickness. After deparaffinization and rehydration, the sections were sequentially stained with hematoxylin and eosin. Histologic changes were evaluated by two independent pathologists, who had no knowledge of the treatment regimen received by each respective animal. The degree of lung injury was scored on a subjective scale ranging from 0 to 3; 0 = absence, 1 = mild, 2 = moderate, and 3 = severe. The ranging scale was used for each of histologic features: congestion, edema, inflammation, and hemorrhage. The final score will be the adding of the single evaluation.

### 2.4. Wet/Dry Weight Ratio

To quantify the magnitude of pulmonary edema, we evaluated lung wet/dry (W/D) weight ratio at 24 hrs after NS or ZYM administration. The harvested wet lung was weighed and then placed in an oven for 24 hrs at 80°C and weighed when it was dried. The ratio of wet lung to dry lung was calculated [13].

### 2.5. BALF Collection

At 24 hrs after administration of ZYM or NS, BALF collection was performed by the methods described previously [14]. The mice were anesthetized with pentobarbital, tracheas were cannulated after exsanguination, and lungs were gently washed with 2 mL of PBS. The amount of exudate was calculated by subtracting the volume injected (2 mL) from the total volume recovered. BALF samples were centrifuged at 500 g at 4°C for 12 mins, and the supernatant was stored at −70°C for subsequent analysis of protein and cytokine levels.

### 2.6. Measurement of Lung MPO Activity

Myeloperoxidase (MPO) activity was measured as an indicator of neutrophil infiltration into the lung tissue as previously described [15]. At 24 hrs after ZYM or NS injection, all animals (*n* = 8 for each group) were sacrificed with pentobarbital. Lungs were obtained and perfused with cold PBS to remove all blood, and homogenated lung supernatants were prepared to detect the activity of MPO. MPO activity was defined by the change in absorbance measured by spectrophotometer at 590 nm and expressed in unit per gram weight of wet tissue. The activity of MPO was measured by using commercial kits purchased from Boster Biotechnology (Wuhan, China).

### 2.7. Measurement of Cytokine Production

At 6 hrs after ZYM or NS injection, the cytokines levels in BALF and lung tissue were measured using commercially available enzyme-linked immunosorbent assay (ELISA) kits (mouse TNF-α, IL-1*β*, and IL-6 ELISA kits are from Boster Biotechnology, Wuhan, China). The optical density (OD) was measured on an ELISA plate scanner. All experiments were performed according to the manufacturers' instructions.

### 2.8. Immunohistochemistry

Immunohistochemistry was performed as previously described [16]. At 24 hrs after ZY or NS injection, the lung tissues were fixed in 10% PBS-buffered formalin, and 5 *μ*m sections were prepared from paraffin-embedded tissues. After deparaffinization, endogenous peroxidase was blocked with 0.3% (volume/volume [v/v]) hydrogen peroxide in 60% (v/v) methanol for 30 mins. The sections were permeabilized with 0.1% (v/v) PBS-buffered Triton X-100 for 20 mins. Incubate the section in 3% (v/v) normal goat serum in PBS for 20 mins to minimize the nonspecific adsorption. Endogenous biotin or avidin binding sites were blocked by sequential incubation for 15 mins with avidin and biotin (BD Biosciences, CA, USA). The sections were then incubated overnight with rabbit anti-iNOS mAb (Santa Cruz, CA, USA, 1 : 500 in PBS, v/v) or with control solutions. A biotin-conjugated specific secondary anti-immunoglobulin G and avidin-biotin peroxidase complex were used to detect the specific labeling. To verify the binding specificity for iNOS, some sections were also incubated with primary antibody only (no secondary antibody) or with secondary antibody only (no primary antibody). In these situations, no positive staining was found in the sections indicating that the immunoreactions were positive in all the experiments carried out.

### 2.9. Western Blot Analysis

The lung tissues were harvested, after mice were killed, and homogenized immediately. Cytoplasmic proteins were extracted from the lungs using Cytoplasmic Protein Extraction Kit (Beyotime Biotechnology, Jiangsu, China) according to the manufacturer's protocol. For extraction of nucleoprotein, lung tissue were homogenized and lysed in the lysis buffer (10 mM Hepes pH 7.9, 1.5 mM MgCl_2_, 10 mM KCl, 0.5 mM DTT, 2% NP-40, and 1 mM PMSF) for 30 min. After that, the lysis buffer was centrifugated at 1,2000 ×g for 15 minutes. Then the supernatant was collected as cytoplasmic protein. Precipitation was washed twice and lysed in the lysis buffer containing Triton X-100 as nucleoprotein. Protein concentrations were determined by BCA protein assay kit. The protein samples were separated by 10% sodium dodecyl sulfate-polyacrylamide gel electrophoresis and were transferred to a polyvinylidene difluoride membrane. The membrane was incubated overnight with antibodies against iNOS (1 : 400), I*κ*B-α (1 : 800), p-I*κ*B-α (1 : 1000), *β*-actin (1 : 1000), NF-*κ*B p65 (1 : 800), and LaminB1 (1 : 1000). The membrane was then incubated with the secondary antibodies (anti-rabbit or anti-mouse IgG peroxidase conjugated, 1 : 10000). The blots were visualized with ECL-Plus reagent (Sigma).

### 2.10. NF-*κ*B DNA Binding Activity Assay

At 6 hrs after ZY or NS injection, nuclear extracts of homogenated lung tissue were prepared. The DNA binding activity of NF-*κ*B in lung tissues was quantified using the TransAM NF-*κ*B p65 transcription factor assay kit (Active Motif, Carlsbad, CA). According to the manufacturer's instructions, all standards and samples were run in duplicate.

### 2.11. Statistical Analysis

Data were expressed as mean ± SEM. Differences between groups were examined for statistical significance using one-way analysis of variance with Student's *t*-test. A *P* value less than 0.05 was considered statistically significant.

## 3. Results

### 3.1. Agmatine Relieves Zymosan-Induced Lung Injury in Mice

**Figure 1:**
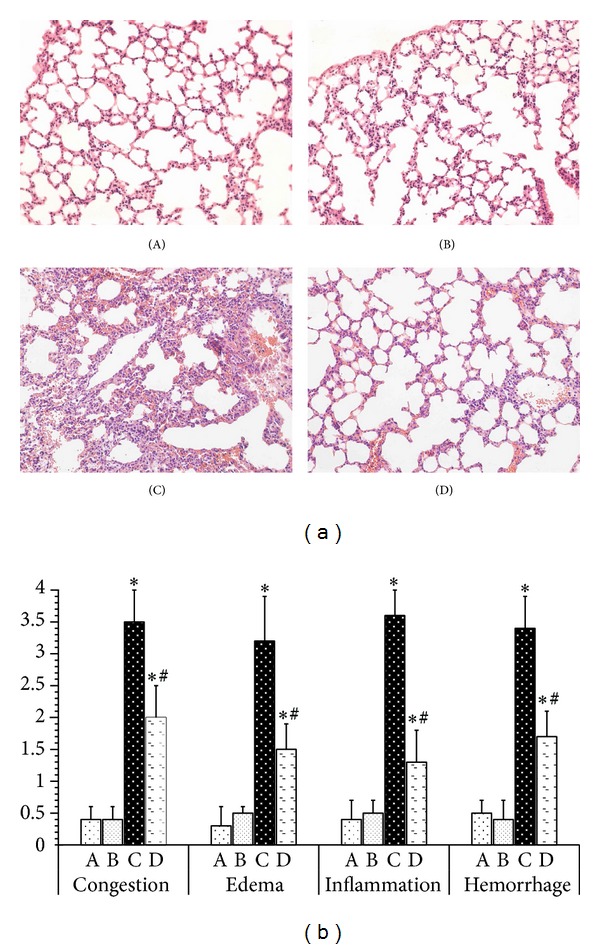
Agmatine relieves Zymosan-induced lung injury in mice. (a) Representative micrographs of H&E staining. Lung tissues in NS group (A), agmatine group (B), Zymosan group (C), and Zymosan + agmatine group (D) were measured. (b) The degree of lung injury was scored by evaluating each of histologic features: congestion, edema, inflammation, and hemorrhage. *P < 0.05 compared with NS group. ^#^ *P* < 0.05 compared with Zymosan group.

Lung injury was characterized by alveolar thickening, infiltration of neutrophils into the lung interstitium, and alveolar space as well as alveolar hemorrhage. As shown in Figure 1(a), the mice in the control group or AGM-treated alone group showed no significant morphologic damages, indicating that intraperitoneal administration with Saline did not induce additional inflammation response in this protocol. However, ZYM-challenged mice appeared to have significant neutrophil infiltration into lung interstitium, alveolar wall thickening, and alveolar hemorrhage. Interestingly, agmatine treatment reduced infiltrated inflammatory cells and improved lung architecture in ZY-challenged mice. A scoring system was used to grade the degree of lung injury by evaluating congestion, edema, inflammation, and hemorrhage. Lung histologic scores significantly increased in ZY-challenged mice (*P* < 0.05) but were reduced by agmatine treatment (*P* < 0.05) (Figure 1(b)).

### 3.2. Agmatine Downregulates Zymosan-Induced TNF-α, IL-1*β*, and IL-6 in Lung and BALF

**Figure 2:**
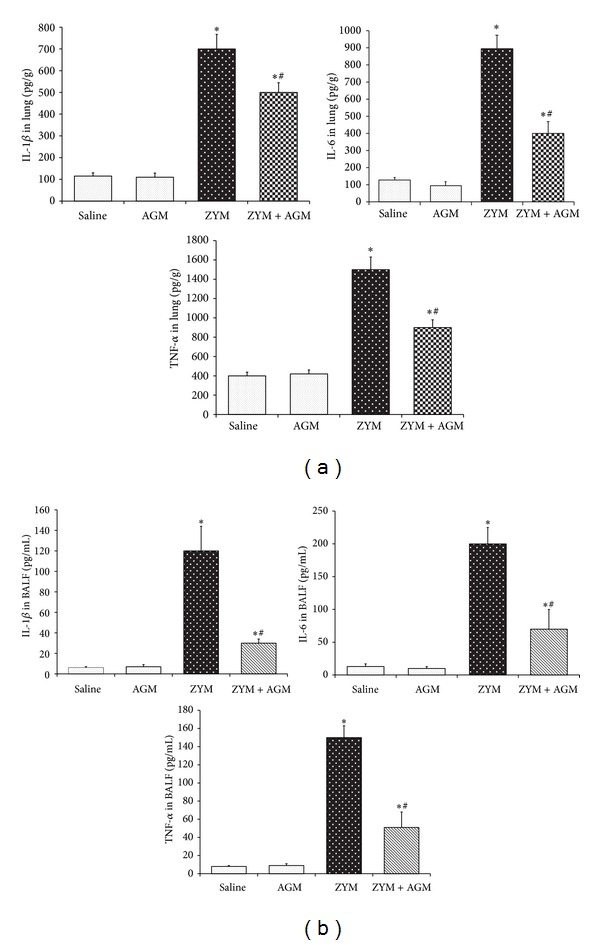
Agmatine downregulates Zymosan-induced TNF-α, IL-1*β*, and IL-6 in lung and BALF. At 6 hr after ZYM or Saline injection, the cytokines levels in lung and BALF tissue were measured using commercially available enzyme-linked immunosorbent assay (ELISA) kits. *P < 0.05 compared with NS group. ^#^ *P* < 0.05 compared with Zymosan group.

To test whether agmatine modulates the inflammatory process through the regulation of secretion of proinflammatory cytokines, we detected the levels of TNF-α, IL-1*β*, and IL-6 in lung and BALF. Six hours after ZYM or Saline injection, the lung and BALF were obtained and measured by ELISA analysis. In the Zymosan group, the concentration of TNF-α, IL-1*β*, and IL-6 in lung (1521.5 ± 128.4, 718.5 ± 67.2, and 917.4 ± 89.5, resp.) and BALF (157.5 ± 13.4, 124.7 ± 23.9, and 197.1 ± 24.3, resp.) increased significantly compared with that of the sham group in lung (421.1 ± 38.4, 114.8 ± 16.4, and 135.2 ± 17.4, resp.) and in BALF (8.1 ± 1.2, 6.5 ± 2.1, and 13.5 ± 4.2, resp.) (all *P* < 0.05). However, in the ZYM + AGM group, the levels of TNF-α, IL-1*β*, and IL-6 in lung (926.2 ± 89.4, 495.5 ± 54.2, and 424.3 ± 74.7, resp.) and BALF (56.7 ± 17.5, 35.02 ± 4.7, and 70.5 ± 33.6, resp.) were significantly lower compared with that of the Zymosan group (all *P* < 0.05) (Figure 2).

### 3.3. Effects of Agmatine on Zymosan-Induced Wet/Dry Weight Ratio, Protein in BALF, and MPO Activity

**Figure 3:**
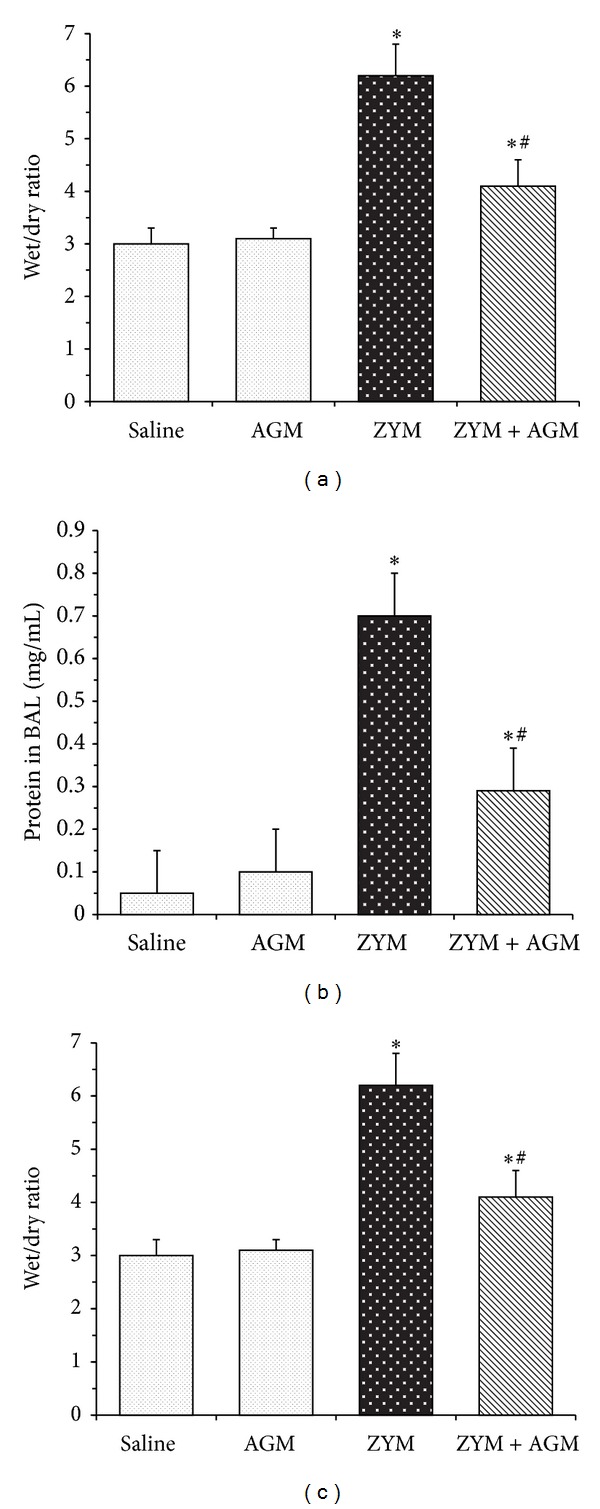
Effects of agmatine on Zymosan-induced wet/dry weight ratio, protein in BALF, and MPO activity. 24 hour after Zymosan challenge, the lung tissues were obtained to detect lung weight and dry ratio (W/D) (a), protein concentration in BALF (b), and MPO activity (c). *P < 0.05 compared with NS group. ^#^ *P* < 0.05 compared with Zymosan group.

The pathogenesis of ALI involves increased permeability of the alveolar-capillary membrane, accumulation of protein-rich fluid in the airspaces, pulmonary edema, and pulmonary infiltration of neutrophils. In our study, twenty-four hour after Zymosan challenge, the lung tissues were obtained to employ lung weight and dry ratio (W/D), protein concentration in BALF, and MPO activity. In the Zymosan group, the W/D ratio (6.2 ± 0.6) was significantly increased compared with that of the Saline group (2.9 ± 0.4; *P* < 0.05). However, the ratio was significantly decreased in the ZYM + AGM group (4.1 ± 0.5; *P* < 0.05) compared with that of the Zymosan group (Figure 3(a)). Besides, the protein concentration in bronchoalveolar lavage fluid (BALF) was also increased in Zymosan group (0.71 ± 0.22) compared with that of the Saline group (0.06 ± 0.01; *P* < 0.05), whereas its level in ZYM + AGM group (0.29 ± 0.11) was significantly lower than that of the Zymosan group (*P* < 0.05) (Figure 3(b)). MPO activity, a biochemical marker of neutrophil infiltration, rose to 25.2 ± 1.8 in the lung of the Zymosan group compared with that of the Saline group (5.2 ± 1.4; *P* < 0.05). Treatment with agmatine resulted in a significant reduction in the lung MPO activity of the ZYM + AGM group (11.0 ± 3.4; *P* < 0.05) compared with that of the Zymosan group (Figure 3(c)).

### 3.4. Agmatine Reduces iNOS Expression in Lung

**Figure 4:**
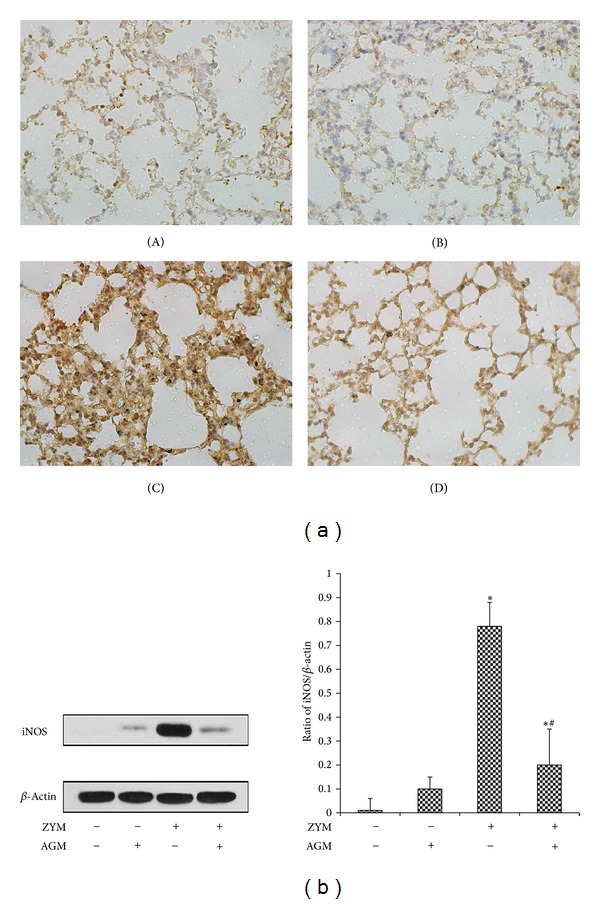
Agmatine decreases iNOS expression in lung. (a) Lung samples were obtained from NS group (A), agmatine group (B), Zymosan group (C), and Zymosan + agmatine group (D) for immunohistochemical staining. (b) Lungs were harvested for observing iNOS expression at 24 hrs after ZYM or NS administration by Western blot. *P < 0.05 compared with NS group. ^#^ *P* < 0.05 compared with Zymosan group.

To understand the iNOS expression in lung, the lung tissues obtained at 24 h after Zymosan administration were detected by immunohistochemistry (Figure 4(a)) and Western blot analysis (Figure 4(b)). A significant increase of iNOS expression in ZYM group was detected compared to that in Saline group by evaluating gray level ratio of iNOS/*β*-actin (0.78 ± 0.12 versus 0.05 ± 0.03, *P* < 0.05). However, agmatine treatment significantly attenuated iNOS expression in the lung compared to that in Saline group (0.23 ± 0.12 versus 0.78 ± 0.12, *P* < 0.05).

### 3.5. Agmatine Inhibits Zymosan-Induced NF-*κ*B Activation and DNA Binding Activity in Lung

**Figure 5:**
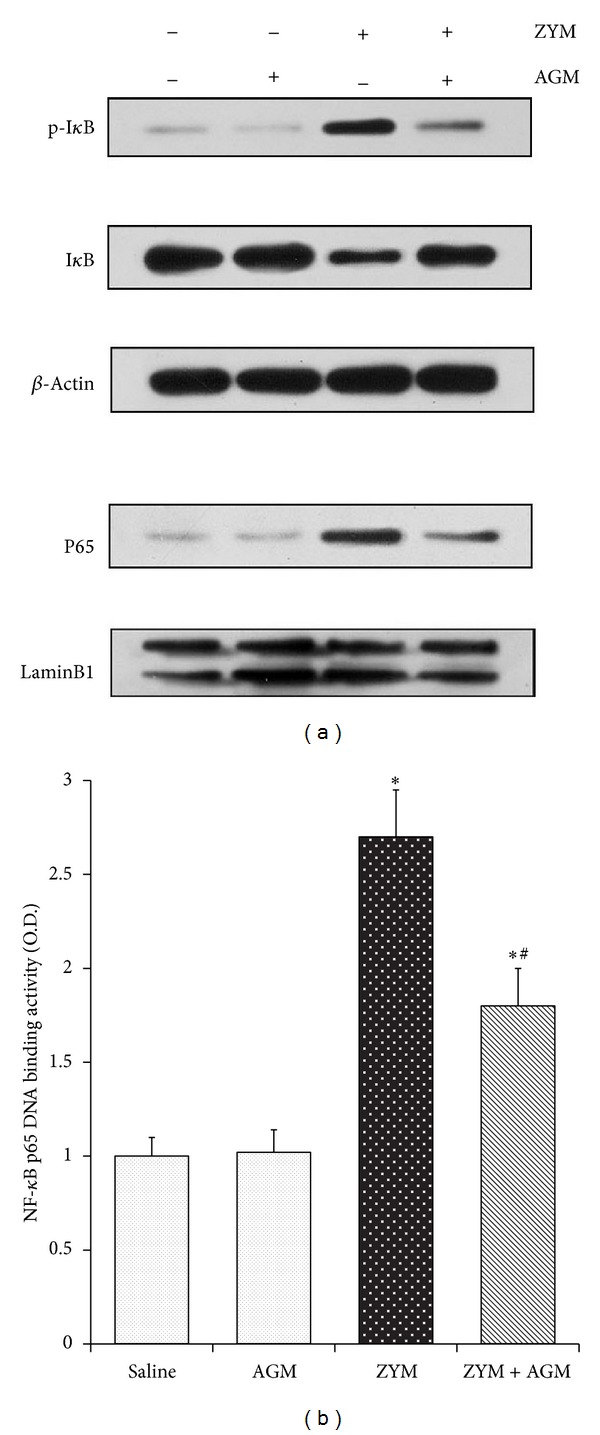
Agmatine inhibits Zymosan-induced NF-*κ*B activation and DNA binding activity in lung. 6 h after ZYM injection with or without agmatine treatments, mice were exsanguinated and their lungs were removed. (a) Western blot was performed to detect p-I*κ*B-α and I*κ*B-α in cytoplasm and NF-*κ*B p65 in nucleus. Expressions of *β*-actin and LaminB1 were shown as loading controls. (b) DNA binding activity of NF-*κ*B p65 was examined by a TransAM p65 transcription factor ELISA kit. Each bar represents the mean ± SD of 8 mice. *P < 0.05 compared with NS group. ^#^ *P* < 0.05 compared with Zymosan group.

Nuclear factor (NF)-*κ*B signaling plays a central role in the initiation and regulation of cellular inflammatory response to bacterial stimuli. Thus, to investigate the mechanisms in which agmatine enhanced lung inflammation resolution, we assessed the effects of agmatine on the degradation of I*κ*B-α and activation of NF-*κ*B by Western blot analysis, and further we detected its effect on DNA binding activity. The mice treated with ZYM exhibited significant degradation of I*κ*B-α in lungs, whereas agmatine treatment at the dose of 200 mg/kg prevented the I*κ*B-α degradation. In contrast, ZYM challenge induced the nuclear translocation of NF-*κ*B p65 subunit, compared with basal group. Agmatine treatment inhibited the nuclear translocation of NF-*κ*B p65 (Figure 5(a)). Furthermore, the Zymosan administration significantly increased NF-*κ*B p65 DNA binding activity in ZYM group compared with that in Saline group (2.70 ± 0.25 versus 1.10 ± 0.12, *P* < 0.05), whereas its activity in ZYM + AGM group was inhibited compared with that in ZYM group (1.82 ± 0.21 versus 2.70 ± 0.25, *P* < 0.05) (Figure 5(b)).

## 4. Conclusion 

Zymosan has been well recognized in the pathogenesis of ALI. Experimental administration of Zymosan, both systemically and intratracheally, has been used to induce neutrophil infiltration and develop pulmonary inflammation in animal models [17, 18]. In the present study, we demonstrated that agmatine protected against ZYM-induced lung inflammation and lung injury in mice. Agmatine treatment inhibited the neutrophil infiltration and lung endothelial permeability in lung of ZYM-challenged mice. Histopathological examination showed that ZYM-induced congestion, edema, inflammation, and hemorrhage in lung were relieved by agmatine. In addition, ZYM induced upregulations of proinflammatory mediators and cytokines in BALF and lung were partially inhibited by agmatine. Furthermore, agmatine treatment inhibited the degradation of I*κ*B-α and subsequent activation and DNA binding activity of NF-*κ*B in lung. These results demonstrate that agmatine exerts a protective effect on ALI via inhibiting NF-*κ*B-mediated inflammatory response.

ALI is characterized by excessive neutrophil infiltration, release of proinflammatory mediators, and loss of vascular barrier integrity [19]. During airway inflammation, neutrophils are the first cells to be recruited and are the predominant cause of tissue damage [20]. Activated neutrophils induce extensive lung inflammation and the destruction of basement membrane and increase the permeability of alveolar capillary membrane [21]. Besides, neutrophils can release damaging mediators, such as cytokines and oxidants, leading to the injury of epithelial-vascular barrier [22]. In the present study, Zymosan-induced mice group caused excessive production of MPO, an enzyme located mainly in the primary granules of neutrophils. Agmatine treatment significantly reduced neutrophil infiltration by determining MPO activity in lung. Furthermore, we detected less protein concentration in BAL and lung W/D weight in mice treated with agmatine, showing the protective effect of agmatine against lung endothelial permeability injury.

Except for neutrophils and the other inflammatory cells, the release of proinflammatory mediators has been reported to be involved in inflammatory cascade [23]. Among them, TNF-α, IL-1*β*, and IL-6 were considered the most important inflammatory mediators in innate immune response. It has been reported that resident alveolar macrophages release TNF-α and IL-1*β* in early phase of ALI in response to ZYM stimulation, resulting in the subsequent inflammatory cascade and tissue injury [24]. It is reported that TNF-α elevates intracellular reactive oxygen species which causes mitochondrial damage or ion exchange dysfunction across the cell membrane [25, 26]. Besides, inhibition of TNF-α was proved to be protective in an animal model of ALI [27]. IL-1*β* also enhances the recruitment of inflammatory cells into airspaces and alters vascular permeability leading to fluid transport and subsequent lung edema formation [28]. During inflammation, elevated TNF-α and IL-1*β* are associated with a poor ALI prognosis [29]. In the present study, the expressions of TNF-α, IL-1*β*, and IL-6 in lung and BALF were markedly induced by ZYM challenge, which was blocked by the treatment of agmatine. Our results confirmed the link between the inflammatory cytokine levels and the extent of lung inflammation, demonstrating the anti-inflammatory effects of agmatine.

Inflammatory activity is mediated by the endogenous free radical NO, which is produced at high levels upon the induction of NO synthase by inflammatory stimulus [30]. In an experimental murine model, ZY administration increases iNOS expression and activity that exacerbates nonseptic shock and leads to cellular and tissue damage including lung injury if unchecked. However, iNOS inhibitors suppress airway inflammation in mice by downregulating proinflammation and chemokine expression that are detrimental to the lung [31]. iNOS-deficient mice also undergo less lung injury after ZY challenge [32]. In the present study, we demonstrated that agmatine treatment significantly reduced iNOS expression and its activity in the lung tissue, and this phenomenon significantly inhibited the inflammatory response. Moreover, NF-*κ*B is a potent regulator of iNOS expression [33], and our results show that agmatine treatment inhibited NF-*κ*B activity as well as iNOS expression in lung. Based on these findings, we speculated that agmatine probably inhibited iNOS expression and activity by blocking NF-*κ*B activation.

It has been reported that NF-*κ*B, a transcription factor, plays a pivotal role in the pathogenesis of immune and inflammatory responses [34]. In experimental animal models of ALI, NF-*κ*B activation is increased [35]. Pharmacological inhibition of NF-*κ*B pathway decreases the production of proinflammatory mediators and protects against endotoxin-induced ALI in animals [36]. Therefore, NF-*κ*B pathway is involved in the pathogenesis of ALI and is known as an important target for anti-inflammatory molecules [37]. Our present study displayed that ZYM obviously enhanced the phosphorylation and degradation of I*κ*B-α. However, agmatine treatment dramatically regulated that this trend, which suggested that suppression of I*κ*B-α activity may be the main reason of agmatine lessening ZYM-initiated pulmonary inflammation in mice. These results suggested that inhibition of NF-*κ*B signaling plays a role in the protective effects of agmatine on ALI.

Agmatine is a cationic polyamine under physiological conditions and thus is thought not to be able to permeate biological membranes, although agmatine can cross the blood-brain barrier when administered peripherally in high doses. In mammalian tissues, agmatine binds to several receptors, including imidazoline, α2-adrenergic, and NMDA glutamate receptor. Sameer et al. reported that agmatine attenuated the acquisition of ethanol conditioned place preference by imidazoline (I_1_ or I_2_) receptors [38]. Bhalla et al. found that agmatine potentiated oxycodone antinociception in mice via an imidazoline I_2_ receptor-mediated mechanism [39]. Besides, agmatine was found to be protective against glutamate-induced necrotic neuronal cell death through NMDA receptor blockade by interacting with a site located within the NMDA channel pore [40]. Therefore, we speculated that agmatine may protect against zymosan-induced acute lung injury by interacting with its endogenous receptors, which will be tested and verified in the next round of experiments.

In summary, we have demonstrated the protective effects of agmatine on ZYM-induced ALI. It is evidenced by alleviating lung inflammation, reducing neutrophil infiltration, decreasing vascular leakage, and proinflammatory cytokine release, inhibiting NF-*κ*B activation and DNA binding activity in ZYM-challenged mice. These results suggest agmatine may be considered as an effective and safe drug for the potential treatment of ALI.
